# Alterations of the Mice Gut Microbiome via *Schistosoma japonicum* Ova-Induced Granuloma

**DOI:** 10.3389/fmicb.2019.00352

**Published:** 2019-03-05

**Authors:** Yanqing Zhao, Shuguo Yang, Bei Li, Wei Li, Jue Wang, Zongyun Chen, Jing Yang, Huabing Tan, Jian Li

**Affiliations:** ^1^Department of Human Parasitology, School of Basic Medical Science, Shiyan, China; ^2^Department of Infectious Diseases, Renmin Hospital, Hubei University of Medicine, Shiyan, China; ^3^Department of Prevention and Control of Schistosomiasis, Jiangsu Institute of Parasitic Diseases, Wuxi, China

**Keywords:** *Schistosoma japonicum*, egg granulomas, gut microbiome, 16S rRNA, operational taxonomic units

## Abstract

Schistosomiasis, also called bilharziasis, is a neglected tropical disease induced by *Schistosoma* spp. that causes hundreds of millions of infections. Although *Schistosoma* ova-induced granulomas commonly cause inflammation, hyperplasia, ulceration, micro abscess formation, and polyposis, the role of the egg granuloma on the gut microbiome remains unclear. To explore the role, gut microbial communities in mice infected with *Schistosoma japonicum* were surveyed. Female C57BL/6 and BALB/c mice were exposed to cercariae of *S. japonicum* for 45 and 65 days and then sacrificed. Intestinal contents and feces were collected, DNA was extracted, and high-throughput 16S rRNA gene-based pyrosequencing was used to provide a comparative analysis of gut microbial diversity. The intestinal mucosal tissues were also examined. Histopathologic analysis demonstrated that the basic structure of the colonic mucosa was damaged by ova-induced granuloma. Regarding the gut microbiome, 2,578,303 good-quality sequences were studied and assigned to 25,278 Operational Taxonomic Units (OTUs) at a threshold of 97% similarity. The average number of OTUs for C57BL/6 and BALB/c were 545 and 530, respectively. At the phylum level, intestinal microbial communities were dominated by Firmicutes, Bacteroidetes, Proteobacteria, and Verrucomicrobia. Infection with *S. japonicum* modified bacterial richness in the fecal associated microbiota. Exposure significantly modified bacterial community composition among different groups. At the phylogenetic levels, LEfSe analysis revealed that several bacterial taxa were significantly associated with the *S. japonicum*-infected mice. The present results suggest that egg granulomas in the intestine influence differentiation of the gut microbial community under pathophysiological conditions. This result suggests that intestinal microbiome-based strategies should be considered for early diagnosis, clinical treatment, and prognosis evaluation of schistosomiasis.

## Introduction

Schistosomiasis, formerly called snail fever, is caused by the genus *Schistosoma*, particularly *S. mansoni, S. haematobium*, and *S. japonicum* (McManus et al., 2018), and it is considered a global neglected tropical diseases (NTDs) (McManus et al., 2018). As the second-most socioeconomically devastating parasitic disease after malaria, schistosomiasis causes hundreds of millions of infections in certain parts of Asia, Africa, and Latin America (Ross et al., 2002) and threatens the animal husbandry economy worldwide.

Eggs are regarded as the main pathogenic factor of schistosomiasis, and ova-granulomas develop in the tissues and organs of human and reservoir hosts (Schwartz and Fallon, 2018). The greatest number of egg granulomas of schistosomiasis develop at locations of maximal egg accumulation, including the intestine, liver and genitourinary tract (Ross et al., 2002). Diarrhea is common, as is occult blood in the feces. Eggs retained in the gut wall induce inflammation, hyperplasia, ulceration, micro abscess formation, and polyposis, but little is known about the relationship between schistosome infection and the composition of the gut microbiome. Fortunately, a recent study showed fecal microbiome differences between *S. haematobium* infected and uninfected children (Kay et al., 2015). Furthermore, two independent studies illustrated that *S. mansoni* infection is associated with an altered gut microbiome (Jenkins et al., 2018; Schneeberger et al., 2018). However, the role that egg granulomas of *S. japonicum* play in the gut microbiome of the mammalian host is still unclear.

To explore the possible role of *S. japonicum* ova-induced granulomas on the gut microbiome, we used a murine model of schistosomiasis, generated by challenge with cercaria of *S. japonicum*. The objectives of this study were: (1) to characterize the microbiota in the gut after damage from *S. japonicum* ova-induced granulomas and (2) to identify bacterial taxa that are associated with *S. japonicum* infection in different mouse strains and different disease phases.

## Materials and Methods

### Parasite Preparation and Animal Studies

Female C57BL/6 and BALB/c mice (24 of each inbred strain) of 6 to 8 weeks of age were randomly divided into the following groups: Control (Ctrl), Acute phase (AP), and Chronic phase (CP). Each group of animals was housed in separate cages, and all mice were maintained in a 12/12 h light/dark cycle at the same temperature (25 ± 1.5°C, 50 ± 5% relative humidity controlled by automatic heating and ventilation devices) and fed standard mouse chow (purchased from HNSJA Co., Ltd., Changsha of China) and pure water (Zhao et al., 2016).

The cercariae of *S. japonicum* (Chinese strain) were obtained from freshwater snails (*Oncomelania hupensis*) that were purchased from the Jiangsu Institute of Parasitic Diseases, Eastern China. In the AP and CP groups, mice were each challenged with 35 ± 1 cercariae. At 42 days post-infection, feces were examined to confirm the infection. At 8 weeks post infection, the host entered the chronic infection stage (Ferrari and Moreira, 2011; Seki et al., 2012; Chen et al., 2017). Under sterile conditions, mice in the Ctrl and AP groups were sacrificed at day 45, and CP mice were sacrificed on day 65.

### Pathologic Analysis

For pathologic analysis, samples of the liver, spleen and intestine were obtained. The tissues were fixed in 10% buffered formalin, embedded in paraffin, sectioned at 4.0 μm, and stained with hematoxylin and eosin for microscopic observation (six tissue samples for each group and three slices for each tissue). All procedures were performed without serious complications. The slides were interpreted under a light microscope (Olympus, Tokyo, Japan). The initial macroscopic diagnosis was made by the parasitologists, and the final diagnosis was confirmed histopathologically.

### Intestinal Content Sampling and DNA Extraction

The feces and intestinal contents were collected under sterile conditions. In brief, on the day of sacrifice, the fresh feces were collected at the time of defecation by scooping the feces into a sterile 15 ml centrifuge tube. For collection of intestinal contents, the intestine was cut into two fragments at the ileocecal junction. The contents were eluted from both fragments of the intestine with 10 ml of sterile normal saline and collected in 15 ml tubes. The tubes were centrifuged at 4,000 rpm for 10 min, and the supernatant was discarded. The feces and intestinal contents from the same mice were mixed and frozen in liquid nitrogen for 15 s and stored at −80°C in the laboratory before shipment.

Samples were packaged with 15 kg dry ice and sent to a company (Novogene Bioinformatics Technology Co., Ltd. in Tianjin, China) where the samples were stored at −80°C until DNA extraction. Total bacterial DNA was extracted from approximately 400–600 mg of each sample using a PowerFecal™ DNA Isolation kit (MO BIO Laboratories, Carlsbad, CA, USA) according to the manufacturer's instructions, and was stored at −80°C before further analysis. The DNA concentration and purity was checked on 1% agarose gel, and the DNA was diluted to a 1 ng/μl working stock.

### Amplification and Sequencing

The 16S rRNA genes of the distinct V4 region (515-806, 392 bp) were amplified using specific primers. Briefly, DNA was amplified by using the 515F and 806R primer set (515F: 5′-GTG CCA GCM GCC GCG GTA A-3′; 806R: 5′- GGA CTA CHV GGG TWT CTA AT-3′), which targets the V4 region of bacterial 16S rDNA, with the reverse primer containing a 6-bp error-correcting barcode unique to each sample. PCR was performed using the Phusion® High-Fidelity PCR Master Mix with GC Buffer containing Taq DNA polymerase premix (New England Biolabs LTD., Beijing, China). The reaction occurred under the following conditions: 94°C for 3 min (1 cycle); 94°C for 45 s, 50°C for 60 s, 72°C for 90 s (35 cycles), and a final step of 72°C for 10 min. The same volume of 1 × loading buffer (containing SYB green) was mixed with the PCR products and underwent electrophoresis on a 2% agarose gel for detection. Samples with a bright main strip between 400 and 450 bp were chosen for further experiments. PCR products were mixed in equi-density ratios and purified by using the QIAquick Gel Extraction Kit (QIAGEN, Dusseldorf, Germany).

Sequencing libraries were generated using the TruSeq® DNA PCR-Free Sample Preparation Kit (Illumina, San Diego CA, USA) following the manufacturer's recommendations, and index codes were added. The library quality was assessed on the Qubit@ 2.0 Fluorometer (Thermo Scientific) and Agilent Bioanalyzer 2100 system. Subsequently, the library was sequenced on an Illumina HiSeq 2500 platform, and 250 bp paired-end reads were generated.

### Data Analysis

Sample paired-end reads were merged by using FLASH V1.2.7 (Magoc and Salzberg, 2011). Quality filtering of the raw sequences was performed with QIIMEV1.7.0. The chimera sequences were detected using the UCHIME algorithm (Edgar et al., 2011) and were removed (Haas et al., 2011). Then, the effective sequences were obtained. Sequences analysis was performed by using UPARSE software package (Edgar, 2013). Sequences with ≥ 97% similarity were assigned to the same OTUs. A representative sequence for each OTU was screened for further annotation. For each representative sequence, the GreenGenes Database (DeSantis et al., 2006) was used based on the RDP classifier Version 2.2 (Wang et al., 2007) algorithm to annotate taxonomic information. Multiple sequence alignments were conducted using the MUSCLE software (Edgar, 2004). OTU abundance information was normalized using a standard of sequence numbers corresponding to the sample with the fewest sequences. Subsequent analyses of alpha diversity and beta diversity were all performed based on this output normalized data.

The alpha diversity in each sample was estimated by Observed species, Chao1, Shannon, and Good-coverage. All these indices in the samples were calculated with QIIME and displayed with R software (Version 2.15.3). Beta diversity analysis was used to evaluate differences in samples regarding bacterial community composition. Beta diversity on (un)weighted UniFrac was calculated by QIIME. Principal Coordinates Analysis (PCoA) was displayed using the WGCNA package, stat packages, and ggplot2 package in R software. Unweighted Pair-group Method with Arithmetic Means (UPGMA) Clustering was performed as a type of hierarchical clustering method to interpret the distance matrix using average linkage and was conducted by QIIME. A Wilcox rank-sum test was used as a significance test of alpha diversity and beta diversity differences between sample groups. Linear discriminant analysis coupled with effect size (LEfSe) was performed to identify the bacterial taxa differentially represented between groups at the genus or higher taxonomy levels.

### Statistical Analysis

Statistical analysis for the gut microbiome in mice was performed as described above. For all other experiments, the data were validated and analyzed using Statistical Package for Social Science (SPSS) for Windows version 17.0 (SPSS Inc., Chicago, IL, USA). To test whether there was a significant difference between two or more groups of sampling units in C57BL/6 and BALB/c mice, Analysis of Similarities (ANOSIM), and Multiple Response Permutation Procedure (MRPP) were used (Sickle, 1997). Statistical significance was defined as a *P*-value less than 0.05.

## Results

### General Information and Pathological Analysis

**Figure 1:**
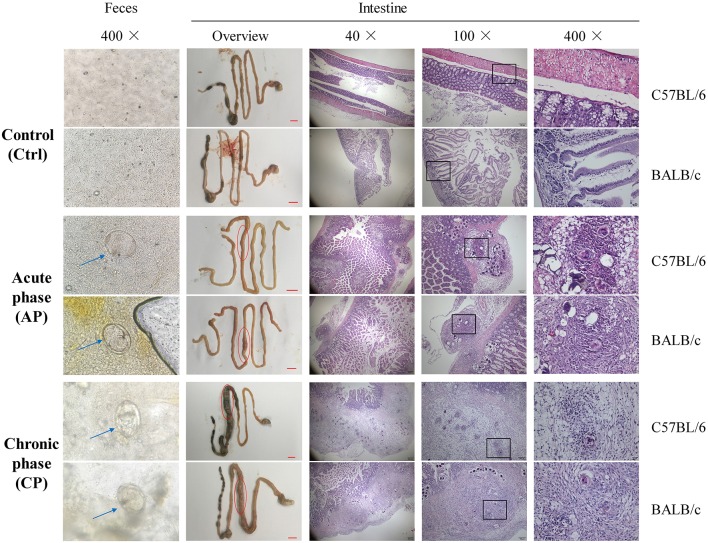
Microscopy visualization and pathological analysis of C57BL/6 and BALB/c mice with *Schistosoma japonicum* infection. For C57BL/6 and BALB/c mice, feces from groups Ctrl and AP at day 45 and CP on day 65 were obtained. The liver, spleen, intestine and feces from groups Ctrl and AP at day 45 and CP on day 65 were obtained. The blue arrow represents an *S. japonicum* egg. The red circle represents a small intestinal lesion. The egg granulomas of *S. japonicum* from the intestinal mucosa are indicated by the black frame.

At Day 0, mice in the AP and CP groups were infected with cercariae of *S. japonicum*. At Day 42, mature eggs containing live miracidium of *S. japonicum* were detected in the feces by microscopy, confirming successful infection (Figure 1). Compared with the Ctrl group, the morphology of the intestine, liver and spleen and the weight of the body, liver, and spleen were dramatically altered for both C57BL/6 and BALB/c infected mice (Figure 1; Supplementary Figure 1). Furthermore, intestinal epithelial edema, inflammation, necrosis, and liver nodules were observed in the infected groups (Figure 1; Supplementary Figure 1). Compared to the AP group of C57BL/6 and BALB/c mice (C57.AP and BA.AP), the intestinal diameter in the CP group of both mouse strains was significantly increased (Figure 1). This usually results from intestinal obstruction, ulcer formation, hemorrhage, and perforation. The main pathomorphological change of the intestinal mucosa was the *S. japonicum* ova-induced granuloma (Figure 1). Further pathological analysis demonstrated that egg granulomas were mainly detected in the submucosa, and only a small number of egg granulomas was found in the muscle layers in C57.AP and BA.AP. In the CP group, the egg granulomatous reactions were not only distributed in submucosa, but numerous egg granulomas had circulated and destroyed muscle layers (Figure 1). Compared to the AP group, more neutrophils infiltrated around the egg granuloma in the CP group (Figure 1).

### Overview of Sequencing Analysis

One sample from the CP group of C57BL/6 mice was discarded due to low quality so that a total of 23 samples (8 Ctrl, 8 AP, 7 CP) from C57BL/6 mice and 24 samples (8 Ctrl, 8 AP, 8 CP) from BALB/c mice were finally collected for 16S rRNA sequencing. In total, 2,730,325 raw sequences from 47 samples were generated, and the number of sequences varied from a minimum of 31,688 sequences obtained in C57BL/6 mice during the chronic phase (CCP4), to a maximum of 69,693 sequences obtained during the chronic phase in BALB/c mice (BCP2, Table 1). The mean number of sequences per sample was 58,092 ± 9,688 (standard deviation, SD). The species accumulation curves indicated that species richness representation in all samples had approached the plateau phase, and it was unlikely that more observed species would be detected without additional samples (Supplementary Figure 2A). Similarly, the rarefaction curves demonstrated that species representation in individual specimens had approached a saturation number of the observed species, and it did not seem as though more OTUs would be obtained with additional sequencing efforts (Supplementary Figure 2B). After sequence trimming, quality filtering and checking for chimeras, a total of 2,578,303 high-quality sequences remained. The number of effective tags varied from a minimum of 26,199 tags obtained in BCP5 to a maximum of 66,089 tags obtained in BCP2, with an average length of 253 bases (Table 1). The GC content in these samples ranged from 52.76 to 55.46%, and the average was 54.26 ± 0.61% (SD). At a threshold of 97% identity, these high-quality sequences were assigned to 25,278 OTUs. Each sample had 54,858 sequences, 538 OTUs, and 469 observed species on average (Table 1). The results showed that the bacteria belonged to 16 phyla, 33 classes, 60 orders, 109 families, and 240 genera.

**Table 1:** Operational taxonomic unit (OTU)-based diversity indexes in mice gut samples during infection.

**Sample name**	**Group**	**Raw PE(#)**	**Raw Tags(#)**	**Clean Tags(#)**	**Effective Tags(#)**	**Base (nt)**	**Q30**	**GC (%)**	**Effective (%)**	**OTUs**	**Observed species**	**shannon**	**chao1**	**Goods coverage**
CC1	C57.Ctrl	62,246	61,755	61,224	59,312	14,985,918	98.98	54.39	95.29	570	486	6.292	525.276	0.997
CC2		53,227	52,890	52,432	50,680	12,811,999	99.00	53.92	95.21	591	519	5.758	636.404	0.996
CC3		40,057	39,756	39,424	37,849	9,566,440	98.84	54.29	94.49	549	506	6.279	577.400	0.997
CC4		57,482	57,138	56,704	54,690	13,826,825	98.97	52.90	95.14	576	494	4.602	559.632	0.997
CC5		64,641	64,143	63,561	61,700	15,591,329	98.92	54.72	95.45	587	509	6.441	567.263	0.997
CC6		65,626	65,124	64,641	62,730	15,856,322	98.97	53.92	95.59	575	496	5.627	539.359	0.997
CC7		68,762	68,300	67,711	66,001	16,675,191	98.97	54.26	95.98	540	460	5.565	531.143	0.997
CC8		53,049	52,704	52,226	50,684	12,808,063	98.96	53.86	95.54	555	505	6.003	602.020	0.996
CAP1	C57.AP	57,887	57,453	56,960	55,080	13,916,102	98.97	53.94	95.15	539	471	5.644	508.143	0.997
CAP2		57,589	57,117	56,602	54,808	13,836,112	98.86	55.15	95.17	577	502	6.349	543.714	0.997
CAP3		67,559	67,036	66,465	64,671	16,334,529	98.89	55.09	95.73	561	476	5.343	522.480	0.997
CAP4		61,722	61,241	60,655	58,670	14,813,426	98.89	54.43	95.06	540	438	5.703	508.288	0.997
CAP5		69,494	69,009	68,480	65,657	16,599,279	98.99	54.94	94.48	500	394	4.582	489.192	0.996
CAP6		52,343	51,960	51,348	50,027	12,695,014	98.95	53.91	95.58	555	516	6.430	581.632	0.997
CAP7		63,355	62,869	62,350	59,812	15,118,445	98.95	54.20	94.41	591	519	6.652	580.158	0.997
CAP8		45,988	45,595	45,139	44,180	11,159,420	98.79	55.46	96.07	511	462	5.205	513.776	0.997
CCP1	C57.CP	62,461	62,036	61,563	58,349	14,750,706	98.99	52.76	93.42	520	436	4.819	507.400	0.997
CCP2		64,117	63,679	63,227	61,538	15,555,415	99.02	54.55	95.98	503	420	4.558	465.310	0.997
CCP3		68,435	68,008	67,543	65,977	16,681,493	99.04	54.52	96.41	480	378	3.517	473.020	0.996
CCP4		31,688	31,426	31,166	30,402	7,682,806	98.8	53.85	95.94	447	405	5.415	438.549	0.998
CCP5		66,501	65,961	65,399	63,865	16,163,855	98.88	55.42	96.04	543	455	5.084	523.018	0.997
CCP6		65,609	64,987	64,437	62,111	15,702,213	98.86	53.69	94.67	569	480	5.868	528.456	0.997
CCP7		58,799	58,334	57,773	56,234	14,216,112	98.82	54.37	95.64	568	498	6.607	552.808	0.997
BC1	BA.Ctrl	61,431	61,035	60,504	59,027	14,911,767	98.93	53.82	96.09	507	424	4.867	476.667	0.997
BC2		69,175	68,624	68,059	66,014	16,687,060	98.90	54.11	95.43	564	485	6.350	550.553	0.997
BC3		55,838	55,480	54,999	53,404	13,503,677	98.96	54.48	95.64	557	475	5.690	531.269	0.997
BC4		34,463	34,177	33,851	32,682	8,262,139	98.81	54.35	94.83	503	466	6.546	498.283	0.998
BC5		68,559	68,067	67,476	65,208	16,484,146	98.93	53.75	95.11	543	479	6.204	516.967	0.997
BC6		67,202	66,742	66,122	64,381	16,277,889	98.88	54.57	95.80	551	482	6.109	518.491	0.997
BC7		60,723	60,318	59,800	58,046	14,673,119	98.92	53.81	95.59	536	461	5.752	530.081	0.997
BC8		53,008	52,685	52,264	50,965	12,881,134	98.99	54.35	96.15	506	455	6.093	540.213	0.997
BAP1	BA.AP	64,636	64,153	63,498	61,339	15,498,643	98.85	53.76	94.90	571	503	6.926	572.081	0.997
BAP2		53,235	52,842	52,374	49,906	12,609,458	98.91	54.29	93.75	571	511	6.497	588.233	0.997
BAP3		62,268	61,778	61,226	57,675	14,564,826	98.91	54.07	92.62	591	522	6.744	570.300	0.997
BAP4		53,458	52,984	52,468	50,340	12,707,679	98.83	54.29	94.17	559	520	6.819	578.000	0.997
BAP5		67,079	66,651	66,034	60,520	15,296,635	98.91	53.62	90.22	558	468	4.587	521.644	0.997
BAP6		62,581	62,093	61,573	55,425	14,012,600	98.95	53.63	88.57	499	421	5.407	487.279	0.997
BAP7		60,239	59,815	59,234	57,919	14,635,439	98.85	54.48	96.15	571	498	6.510	541.596	0.997
BAP8		50,375	49,933	49,497	47,460	11,996,688	98.91	54.76	94.21	567	522	6.243	588.750	0.997
BCP1	BA.CP	34,534	34,279	33,957	33,028	8,344,957	98.77	55.35	95.64	494	455	5.671	484.057	0.998
BCP2		69,693	69,233	68,638	66,089	16,709,132	98.97	52.77	94.83	487	420	4.053	515.136	0.996
BCP3		55,751	55,309	54,806	53,346	13,476,296	98.90	54.86	95.69	504	438	6.555	474.849	0.998
BCP4		58,121	57,687	57,190	55,061	13,914,775	98.96	54.44	94.74	473	408	5.030	442.528	0.998
BCP5		40,336	38,617	26,718	26,199	6,617,591	98.71	54.52	64.95	425	390	5.284	436.406	0.998
BCP6		54,409	54,016	53,599	52,406	13,247,321	98.87	54.92	96.32	542	469	5.091	529.768	0.997
BCP7		63,040	61,192	60,283	57,207	14,560,230	98.71	54.19	90.75	524	465	5.587	522.130	0.997
BCP8		51,534	51,156	50,678	49,629	12,539,299	98.79	54.61	96.30	528	485	6.615	555.660	0.997
Total		2,730,325	2,707,387	2,671,878	2,578,303	651,759,514	4,648.43	2,550.34	4,430.89	25,278	22,047	269.573	24,845.382	46.860
Max		69,693	69,233	68,638	66,089	16,709,132	99.04	55.46	96.41	591	522	6.926	636.404	0.998
Mini		31,688	31,426	26,718	26,199	6,617,591	98.71	52.76	64.95	425	378	3.517	436.406	0.996
Average		58,092	57,604	56,848	54,858	13,867,224	98.90	54.26	94.27	538	469	5.736	528.625	0.997
SD		9,688	9,669	10,216	9,776	2,471,997	0.08	0.61	4.64	39	39	0.788	43.734	0.000

*C57 and BA represent C57BL/6 and BALB/c mice, respectively; Ctrl, AP and CP represent Group Control, Acute phase and Chronic phase, respectively; Max, Mini and SD stand for maximum value, minimum value and Standard deviation, respectively*.

In total, the trends in OTU numbers from the Ctrl to the CP group were different between C57BL/6 and BALB/c mice. For C57BL/6 mice, the OTU number rapidly decreased immediately after infection compared to the Ctrl (Table 1). In contrast, the OTU trends in BALB/c mice increased to a peak in AP and then decreased to a valley in CP. Detailed characteristics of each sample are listed in Table 1. Venn diagram was created to compare the similarities and differences among the communities in the different groups and samples (Supplementary Figure 3). For individual samples, the maximum, minimum and mean unique OTUs were 36 in BC2, 3 in CCP3, and 15, respectively (Supplementary Figure 3).

### Bacterial Community Composition and Structure Succession Analysis

**Figure 2:**
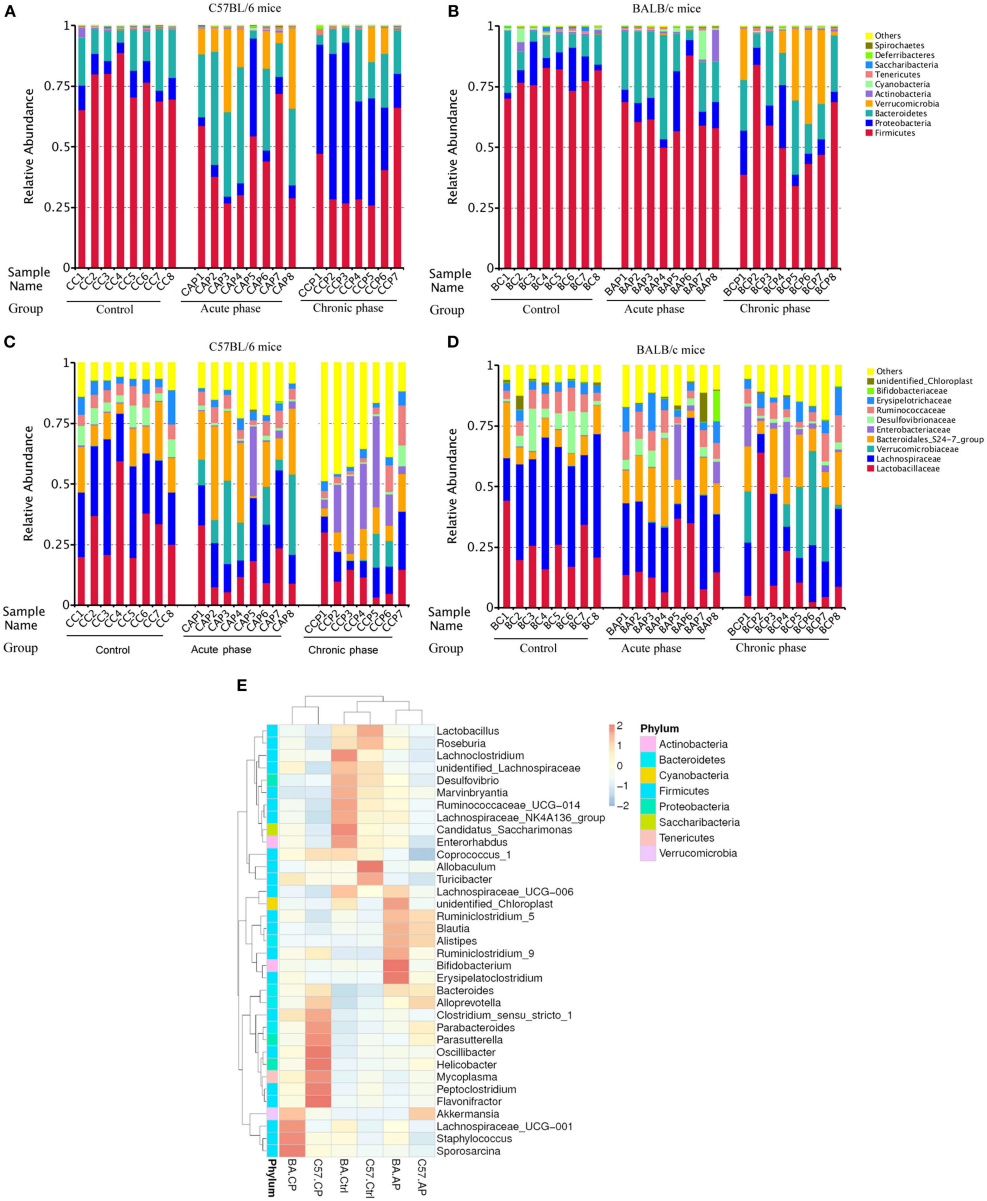
Variation of bacterial community structure. The relative abundance of bacterial phyla and families present in the gut of C57BL/6 and BALB/c mice. Data shown represent the top 10 most abundant phyla and families, whereas low abundance and unclassified OTUs were grouped in “Other.” Sample names refer to samples as described in Table 1. **(A)** Bacterial community structure variation at the phylum level in C57BL/6 mice; **(B)** Bacterial community structure variation at the phylum level in BALB/c mice; **(C)** Bacterial community structure variation at the family level in C57BL/6 mice; **(D)** Bacterial community structure variation at the family level in BALB/c mice. **(E)** Taxonomic heatmap of different groups at the genus level. Characteristic relative abundances of various genera present in the Control (C57.Ctrl, BA.Ctrl), Acute phase (C57.AP, BA.AP), and Chronic phase (C57.CP, BA.CP). The portrait and landscape stand for different group and species annotation information, respectively. The left pattern indicates a clustering tree at the level of genus. The above profile displays clustering trees among different groups. Each column represents a unique subject.

At the phylum level, the intestinal microbial communities in all samples were dominated by Firmicutes (58.87%), Bacteroidetes (18.67%), Proteobacteria (13.80%), and Verrucomicrobia (6.05%) (Figures 2A,B). For individual groups, the dominant phylum had various patterns. Verrucomicrobia was significantly more abundant in C57.AP than in BA.AP (*P* = 0.011). In contrast, Firmicutes was significantly more abundant in the AP group of BALB/c than in C57BL/6 mice (*P* = 0.020). Analogously, Proteobacteria were present at a > 4-fold higher abundance in C57.CP than in BA.CP (*P* = 0.002). In contrast, Verrucomicrobia were present at a > 4-fold higher abundance in BA.CP than in C57.CP (*P* = 0.060). Several of the most abundant taxa from the Ctrl and CP groups gradually increased, particularly in BALB/c mice (Figure 2B). However, the percentage of dominant taxa was dramatically decreased during infection. As the dominant taxon, the abundance of Firmicutes gradually decreased following infection. The abundance of Bacteroidetes dramatically increased on day 45 post-infection, but then decreased from day 65 onwards. The abundance of Proteobacteria gradually increased on day 45 and dramatically increased on day 65 post-infection in C57BL/6 mice (Figure 2A), while the abundance of Proteobacteria throughout the infection had little change in BALB/c mice (Figure 2B).

For all 47 samples, the most common families were Lachnospiraceae (24.3%), Lactobacillaceae (20.0%), and Bacteroidales_S24-7_group (14.2%) (Figures 2C,D). Ruminococcaceae (6.0%), Verrucomicrobiaceae (6.0%), Enterobacteriaceae (5.7%), Erysipelotrichaceae (4.6%), Desulfovibrionaceae (3.7%), and Bacteroidaceae (2.2%) were subdominant families. The dominant families in the Ctrl, AP, and CP groups of C57BL/6 and BALB/c mice are shown in Figures 2C,D, respectively. Lactobacillaceae and Lachnospiraceae gradually decreased from the Ctrl group to the CP group for both mouse strains. The Bacteroidales_S24-7_group increased in abundance for these two strains and became the most abundant and the top two taxa on day 45 but decreased on day 65. Interestingly, the abundance of Ruminococcaceae changed only slightly from the Ctrl group to the CP group for different mouse strains. Surprisingly, the low abundance of Verrucomicrobiaceae in C57.Ctrl (0.08%) was dramatically increased to 15.43% in C57.AP and subsequently decreased to 3.68% in C57.CP (Figure 2C). For BALB/c mice, the abundance of Verrucomicrobiaceae rapidly increased to 16.43% on day 65 post-infection from 0.07% in BA.AP (Figure 2D).

Hierarchical clustering based on the abundance profile of the genera showed that the two strains of mice tended to group together under the same experimental conditions (Figure 2E). Interestingly, non-infected control, and acute phase groups clustered together, whereas on the left of the heatmap, a separation was observed between the AP and CP groups. At the genus level, several taxa showed significant differences among the six groups. In Figure 2E, the most common genus in each group is shown. The gut microbiota in C57.CP was characterized by higher amounts of *Oscillibacter, Helicobacter, Flavonifractor*, and *Peptoclostridium*, and decreased amounts of *Lachnospiraceae_NK4A136_group*. Compared with BA.Ctrl, OTUs belonging to *Bifidobacterium* and *Erysipelatoclostridium* were observed more frequently after infection in BA.AP, whereas *Sporosarcina, Staphylococcus*, and *Lachnospiraceae_UCG-001* were more abundant in the chronic phase BA.CP.

### Comparison of Bacterial Community Within Groups

**Figure 3:**
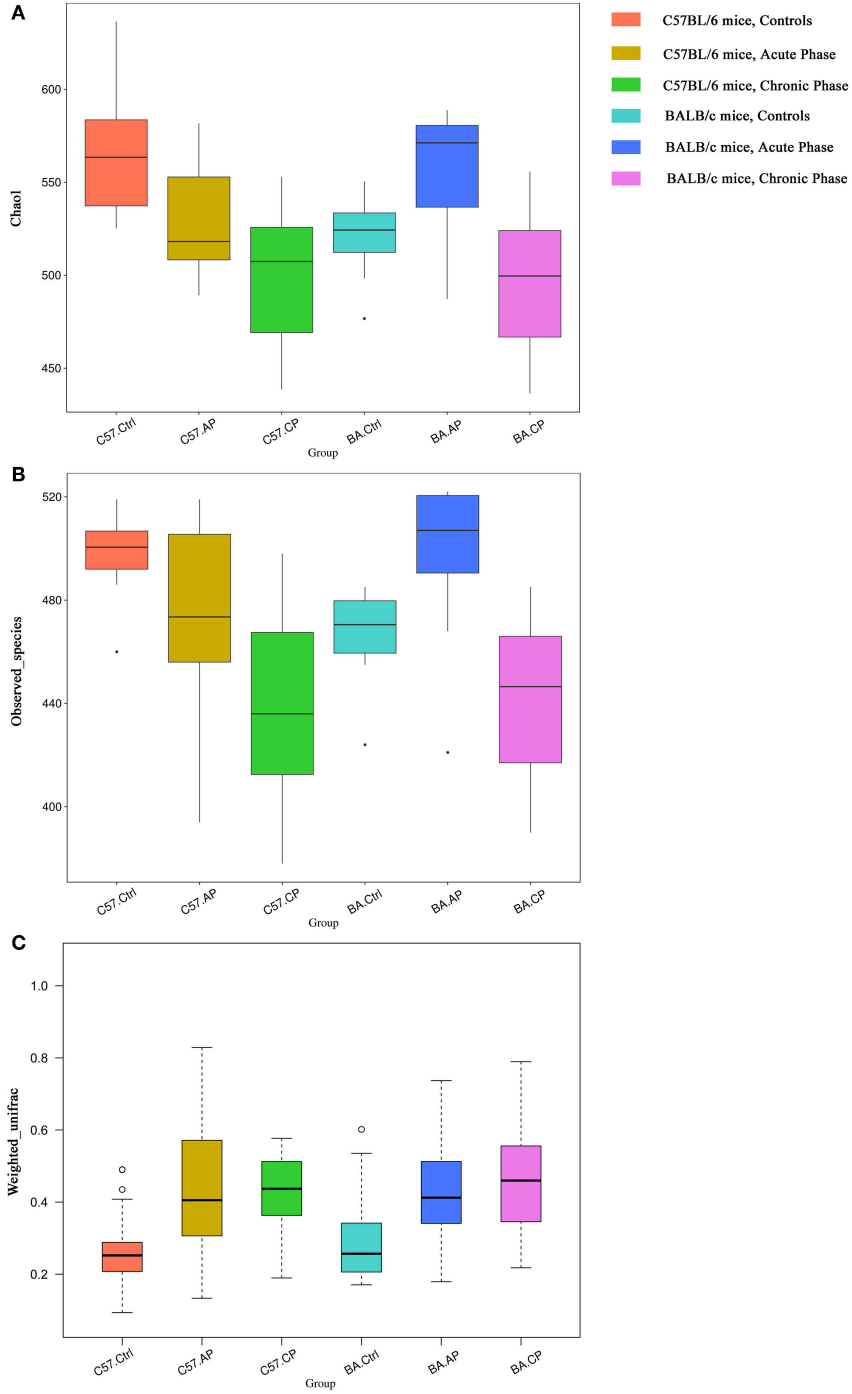
Bacterial community comparison of different groups between uninfected mice and infected mice. Outliers were plotted as individual points. **(A)** Alpha diversity analysis based on the Chao1 index among groups in different mouse strains. **(B)** Alpha diversity analysis based on the observed species index among groups in different mouse strains. **(C)** Beta diversity analysis based on weighted UniFrac in different mouse strain.

Alpha diversity was calculated, including Chao1, good coverage, observed species, and Shannon index (Table 1). The trends of alpha diversity metrics for gut samples from the Ctrl to CP were different between different mice strains. For C57BL/6 mice, OTU numbers, observed species, and Shannon and Chao1 index values declined from group C57.Ctrl to C57.CP. In contrast, these values increased in BA.AP and finally decreased thereafter in BA.CP. The results showed that the interquartile range of species diversity was increased from the Ctrl to AP and CP in both C57BL/6 and BALB/c mice (Figures 3A,B). Outliers were only detected in group C57.Ctrl, BA.Ctrl, and BA.AP (Figure 3B).

For Chao1 analysis (Figure 3A), C57.Ctrl samples had a significantly higher value than C57.AP and C57.CP (*P* = 0.046, *P* = 0.001; Wilcox rank-sum test). For BALB/c mice, BA.AP samples had a significantly higher Chao1 value than BA.Ctrl and BA.CP, respectively (*P* = 0.044 and *P* = 0.002, respectively; Wilcox rank-sum test). For observed species analysis (Figure 3B), C57.Ctrl samples had a significantly higher value than C57.CP (*P* = 0.001; Wilcox rank-sum test). For BALB/c mice, BA.AP samples had a significantly higher value than BA.Ctrl and BA.CP (*P* = 0.017 and *P* = 0.001, respectively; Wilcox rank-sum test). For Shannon diversity, a significant difference was only seen between AP and CP samples of BALB/c mice (*P* = 0.048; Wilcox rank-sum test).

### Comparison of Bacterial Community Among Groups

Based on weighted UniFrac (Figure 3C), the beta diversity for gut samples from the Ctrl to CP were similar in both C57BL/6 and BALB/c mice. The values were increased from group C57.Ctrl to C57.CP. Groups C57.AP and C57.CP had a statistically significant increase in beta diversity compared with C57.Ctrl samples (*P* = 2e-06, *P* = 1.2e-05; Wilcox rank-sum test). BA.Ctrl samples had a significantly lower beta diversity than BA.AP and BA.CP (*P* = 0.0001 and *P* = 2e-06, respectively; Wilcox rank-sum test). This suggested that the range of beta diversity was more heterogeneous in the infected group than in the uninfected control in both C57BL/6 and BALB/c mice. In particular, the degree of dispersion in groups C57.AP and BA.CP was strongly influenced. Outliers were detected in groups C57.Ctrl and BA.Ctrl.

**Figure 4:**
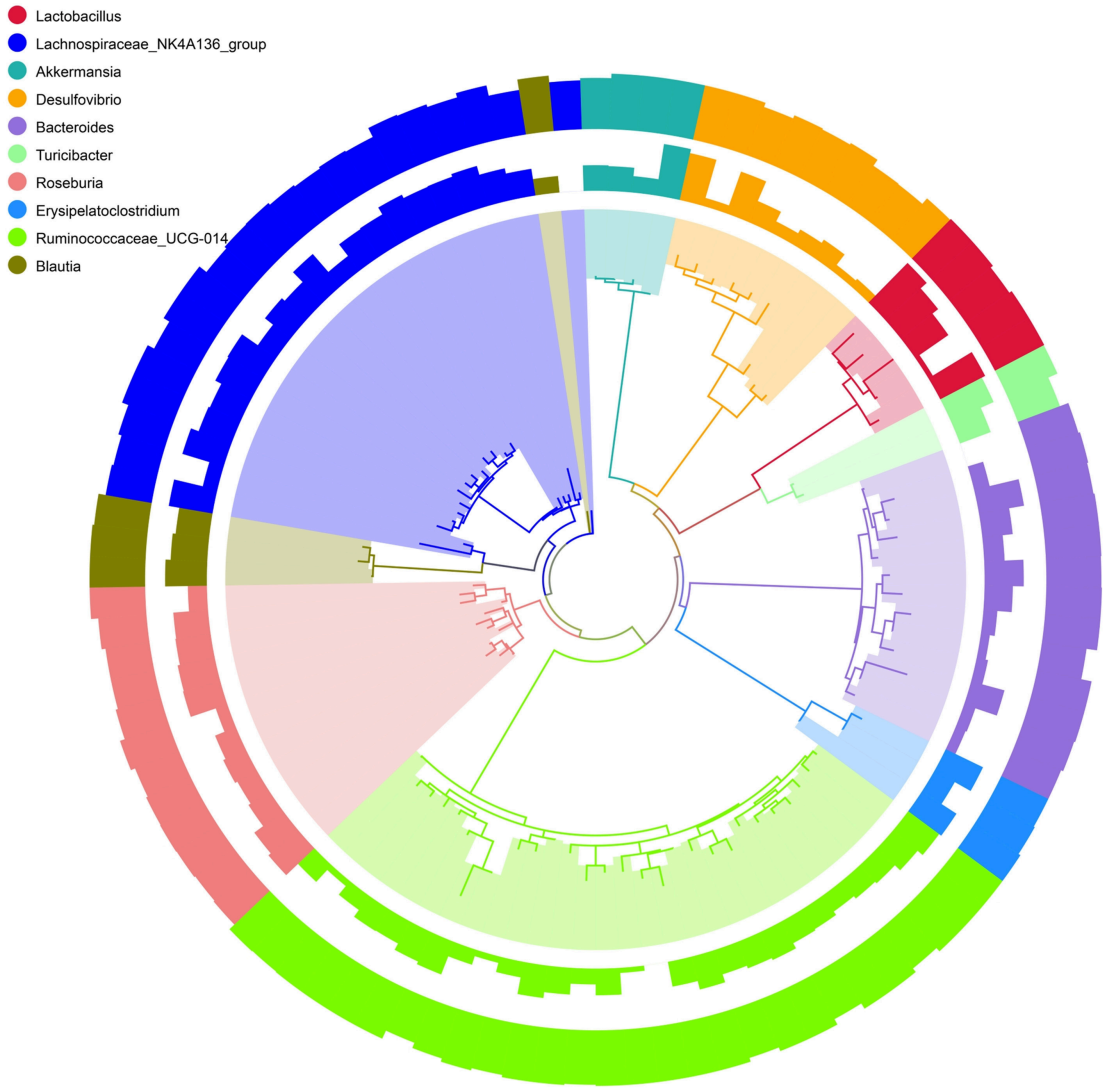
Phylogenetic relationships and Species annotation of Operational Taxonomic Units (OTUs). The inner band shows genera colored by OTUs, the next band shows the relative abundance of OTUs, and the outer band shows the annotation reliability distribution of OTUs. Overall abundance and the magnitude of the difference among genera are indicated by bars.

To better visualize the OUT diversity of mouse gut bacteria with a broader evolutionary context in these two strains, a maximum likelihood phylogeny of the top 10 species (relative abundance of OTUs) was constructed. In Figure 4, a total of 101 OTUs were identified as 10 different species. Among these OTUs, 28 were classified as Ruminococcaceae_UCG-014 and 21 as Lachnospirace_NK4A136_group, which were differently distributed in the groups of C57BL/6 and BALB/c mice. Most OTUs occurred in all groups with different abundances.

**Figure 5:**
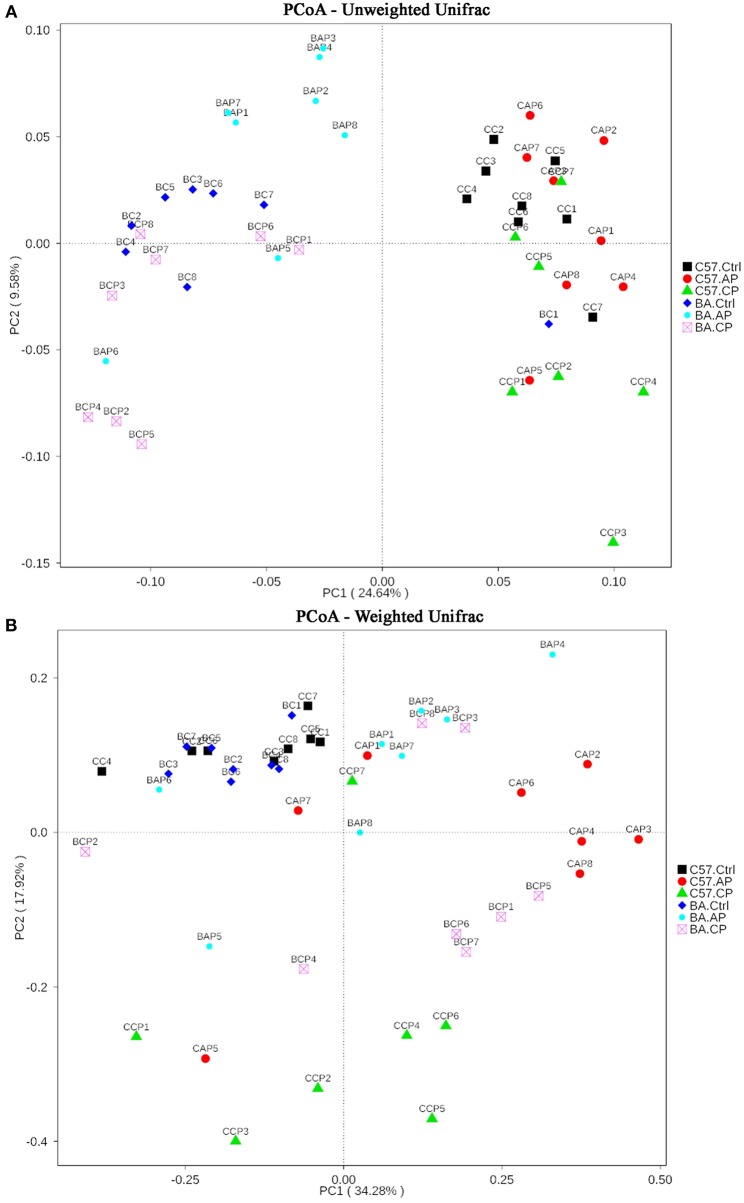
Two-dimensional principal coordinates analysis (PCoA) plot of unweighted **(A)** and weighted **(B)** UniFrac distance matrices for both intestinal contents and feces samples from C57BL/6 and BALB/c mice during infection. The bacterial community of the feces and intestinal contents from C57BL/6 mice in group C57.Ctrl, C57.AP, and C57.CP and from BALB/c mice in group BA.Ctrl, BA.AP, and BA.CP are shown. Sample names refer to samples as described in Table 1.

On the PCoA plot, each symbol represents the gut microbiota of one group (Figure 5). It is noteworthy that the microbiotas from the Ctrl group to the CP group were distinct between C57BL/6 and BALB/c mice (Figure 5A). The relationships between community structures revealed by PCoA were further tested by comparing within-group weighted UniFrac distances in C57BL/6 and BALB/c mice (Figure 5B). Consistent with the PCoA plot, the between-group distances were significantly higher than the within-group distances (ANOSIM, MRPP, *P* ≤ 0.01) for each group (Supplementary Tables 1, 2). These data suggest that the gut microbial community structures between the two strains of mice were significantly different. Additionally, the 47 samples were clustered by UPGMA with an unweighted UniFrac matrix. It showed that C57BL/6 and BALB/c mice were obviously clustered into two independent clusters at the phylum level (Supplementary Figure 4). In each cluster, the main samples in Ctrl, AP, and CP groups were also clustered into subclusters.

### Potential Biomarker Discovery

**Figure 6:**
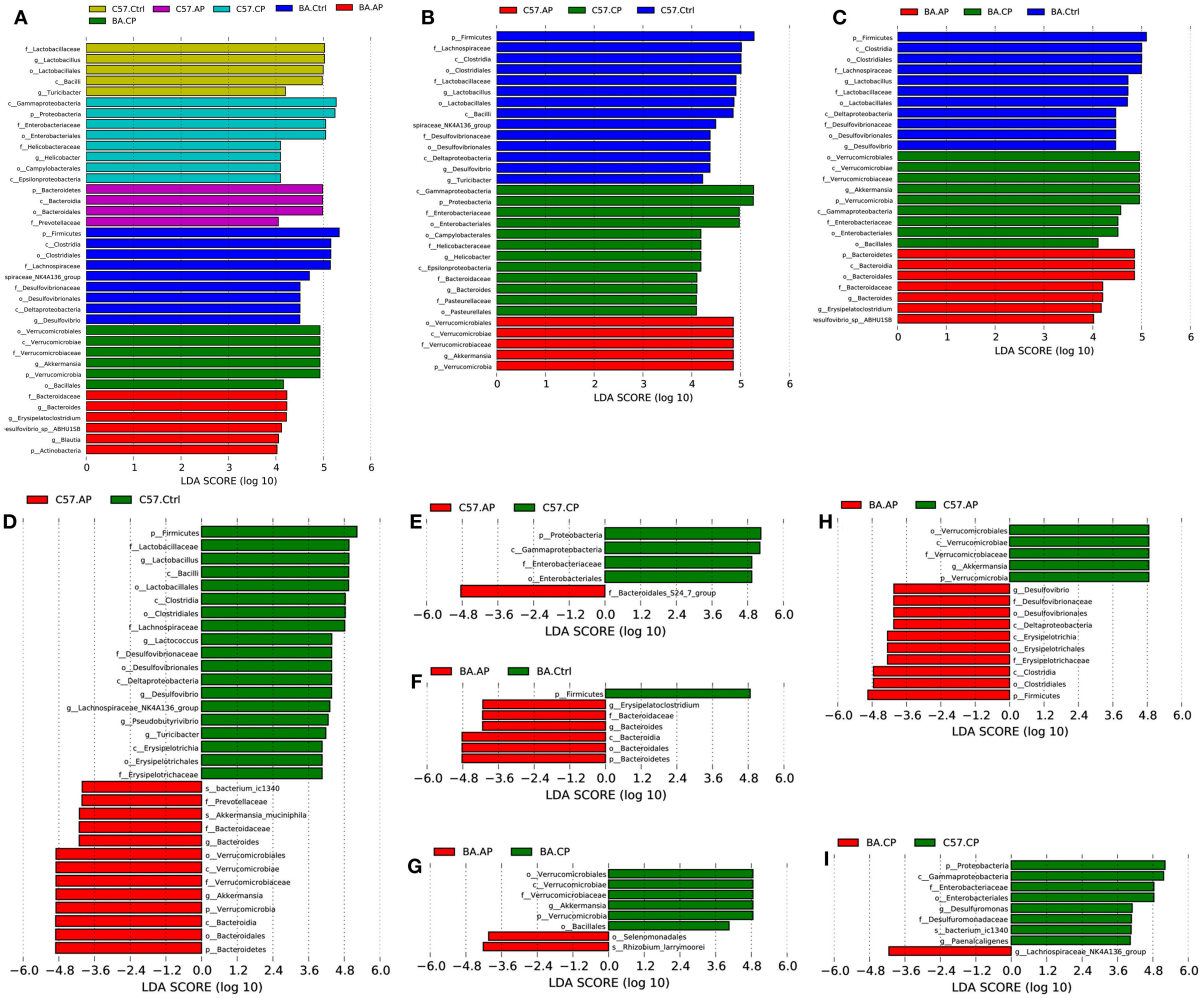
LEfSe identified the most differentially abundant taxa in different groups of C57BL/6 and BALB/c mice. Only taxa meeting an LDA significance threshold >4 are shown in the figures. **(A)** Histogram of linear discriminant analysis (LDA) score distribution among the six groups; **(B)** Histogram of LDA score distribution among three groups of C57BL/6 mice; **(C)** Histogram of LDA score distribution among three groups of BALB/c mice; **(D)** Histogram of LDA score distribution between the C57.Ctrl and C57.AP group; **(E)** Histogram of LDA score distribution between the C57.AP and C57.CP group; **(F)** Histogram of LDA score distribution between the BA.Ctrl and BA.AP group; **(G)** Histogram of LDA score distribution between the BA.AP and BA.CP group; **(H)** Histogram of LDA score distribution between the C57.AP and BA.AP groups. **(I)** Histogram of LDA score distribution between the C57.CP and BA.CP groups.

**Figure 7:**
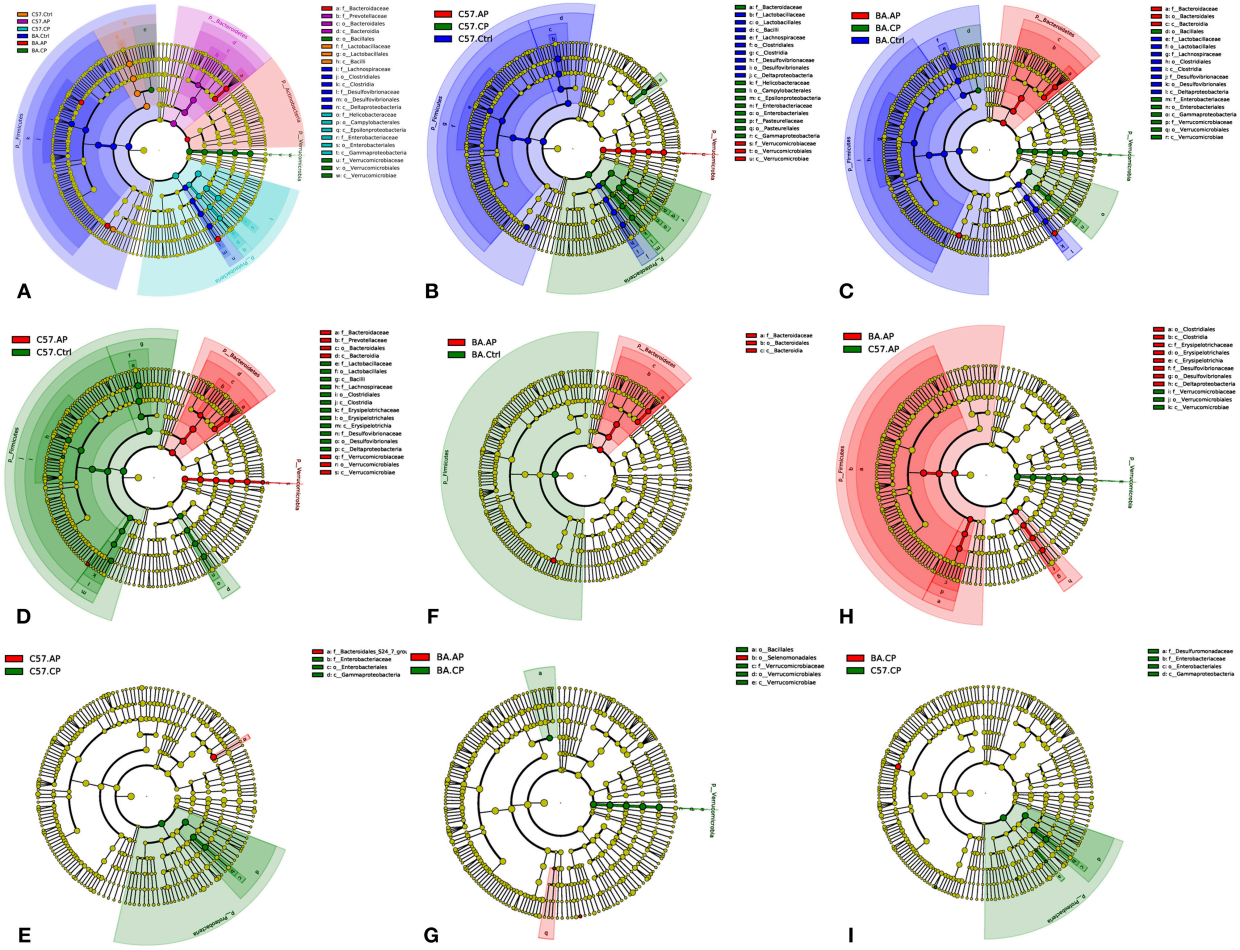
Cladogram of the most differentially abundant taxa in different groups of C57BL/6 and BALB/c mice. **(A)** Cladogram of the most differentially abundant taxa in six groups of C57BL/6 and BALB/c mice; **(B)** Cladogram of the most differentially abundant taxa in different groups of C57BL/6 mice; **(C)** Cladogram of the most differentially abundant taxa in different groups of BALB/c mice; **(D)** Cladogram of the most differentially abundant taxa between C57.Ctrl and C57.AP; **(E)** Cladogram of the most differentially abundant taxa between C57.AP and C57.CP; **(F)** Cladogram of the most differentially abundant taxa between BA.Ctrl and BA.AP; **(G)** Cladogram of the most differentially abundant taxa between BA.AP and BA.CP; **(H)** Cladogram of the most differentially abundant taxa between C57.AP and BA.AP; **(I)** Cladogram of the most differentially abundant taxa between C57.CP and BA.CP.

To identify bacterial taxa that were significantly different between groups of C57BL/6 and BALB/c mice, LEfSe was performed on the taxa. It showed that the bacterial taxa were differentially represented among the different groups of C57BL/6 and BALB/c mice (Figure 6). The potential biomarkers at different taxonomic levels in the groups of C57BL/6 and BALB/c mice were determined (Figure 7). At the genus level, the biomarker with a significant difference between C57.AP and the other two groups in C57BL/6 mice was *Akkermansia*; the biomarkers showing significant differences between C57.CP and the other two groups in C57BL/6 mice were *Helicobacter* and *Bacteroides*. Several genera, including *Lactobacillus, Desulfovibrio*, and *Turicibacter*, were associated with the non-infected control in C57BL/6 mice (Figure 6B). In BALB/c mice, bacteria from the genera *Bacteroides* and *Erysipelatoclostridium* were identified as markers of BA.AP. The genus *Akkermansia* was a marker of BA.CP. The genera *Lactobacillus* and *Desulfovibrio* were enriched in BA.Ctrl (Figure 6C).

## Discussion

In this study, the gut microbial structure in C57BL/6 and BALB/c mice with *S. japonicum* ova-induced granulomas was studied. Both species accumulation curves and rarefaction curves gained from the OTU counts approximate the saturation level, indicating a relatively perfect coverage of the total and individual ecosystem diversity. Based on the alpha diversity analysis, we observed that the richness and diversity of the intestinal microbiota in fecal samples from both C57BL/6 and BALB/c mice had been noticeably changed after *S. japonicum* infection. The beta diversity analysis showed a significantly higher beta diversity in the infected group compared to the uninfected group of these two mouse strains. Consistent with this notion, an overall reduction in alpha diversity and a significant increase in beta diversity were found in the gut microbiota of *S. mansoni*-infected mice when compared to uninfected controls (Jenkins et al., 2018). A loss of microbial diversity in the intestine is also shown in several human intestinal and extraintestinal disorders, including inflammatory bowel disease (IBD), colorectal cancer (CRC), chronic liver diseases, type 2 diabetes, and asthma (Tilg et al., 2018). In the present study, the core gut microbiome at the phylum level in uninfected and infected mice was dominated by Firmicutes, Bacteroidetes and Proteobacteria, which are commonly found in the mouse model (Zheng et al., 2016a,b; Deng et al., 2018; Kim et al., 2019). Significant differences were found before and after infection. *S. japonicum*-treated mice had a significantly different microbiome composition compared to control groups of the two mouse strains based on beta diversity analysis. These findings indicated that *S. japonicum* ova-induced granulomas obviously influenced the microbial composition pattern in mice. This is consistent with a previous study in mice demonstrating the effect of helminth infection on the gut microbiota (Su et al., 2018). However, one study suggested that neither a *S. mansoni* infection nor praziquantel administration in children triggers a significant effect on the microbial composition, although some subtle modifications in the gut microbiome were observed (Schneeberger et al., 2018). The composition of the gut microbiome, especially in human beings, which is not only influenced by infections, but also by host genetic variation and environmental factors, such as nutrition, may explain this paradox (Kau et al., 2011; Blekhman et al., 2015).

Current data demonstrate that the relative abundance of dominant phyla and families show notable variation during the process of infection. Specifically, *S. japonicum* infection is connected with a relative decrease of Firmicutes in the feces compared with uninfected C57BL/6 mice. In contrast, *S. japonicum* cercaria exposure is related to an obvious relative increase of Bacteroidetes and Proteobacteria in the feces compared to untreated C57BL/6 mice. These changes in the abundance of Bacteroidetes and Firmicutes are also detected after human catestatin treatment in C57BL/6 mice (Rabbi et al., 2017). The altered gut microbial composition in mice associated with intestinal inflammation is quite similar to humans with IBD. The patients with IBD also have a comparatively low abundance of Firmicutes and relatively high abundance of Bacteroidetes (Hansen et al., 2010). In the members of the Firmicutes phylum, a loss of *Faecalibacterium prausnitzii* is correlated with an increased risk of postoperative recurrence of ileal Crohn's disease (Quévrain et al., 2016). In the phylum Bacteroidetes, *Bacteroides fragilis* toxin is associated with IBD, particularly in patients with active disease (Prindiville et al., 2000). However, the same variations, including the relative abundance of Firmicutes, Bacteroidetes, and Proteobacteria, are not observed in the feces of BALB/c mice. It demonstrates there is a considerable difference in the gut microbiome between the two mouse strains after *S. japonicum* infection. Moreover, similar results obtained from the PCoA and UPGMA dendrogram demonstrate that the gut microbial community structures after infection were significantly different between C57BL/6 and BALB/c mice. C57BL/6 mice have been a proposed model of *S. japonicum* schistosomiasis for insight into immunological disease process, such as granulomatous inflammation, due to their high level of inflammatory reactions (Mitchell et al., 1981; Hirata et al., 1993). Thus, C57BL/6 mice may be a useful rodent model for further studies of the schistosomal gut microbiome.

Several high-abundance phyla and families, such as Verrucomicrobia and Enterobacteriaceae, should not be ignored. In several diseases involving inflammation in the gut, including IBD and CRC, Enterobacteriaceae are one of the most commonly overgrown symbiotic bacteria (Zeng et al., 2017). Imbalances in Enterobacteriaceae are associated with IBD and CRC. As a common bacterial phylum detected in soil, Verrucomicrobia is also found in the marine environment (Freitas et al., 2012). However, little is known about its role in the murine gut. To address this issue, the microbial community composition from the *S. japonicum* infected mice was analyzed. Subsequently, Verrucomicrobia was detected as the dominant bacterial phylum in C57.AP and BA.CP. Consistently, a significant expansion in populations of *Verrucomicrobiaceae* (species *Akkermansia muciniphila*) was associated with *S. mansoni* infection (Jenkins et al., 2018). A similar result was found in humans following wide-spectrum antibiotic treatment (Dubourg et al., 2013). However, the role of this recently characterized phylum remains unclear. The current findings offer more insights into various host intestinal microbiomes after *S. japonicum* infection and demonstrate that microbiome analysis is potentially powerful for early accurate diagnosis of schistosomiasis and understanding of the pathogenic mechanism of disease progression.

As a broad-spectrum anthelmintic, praziquantel works effectively on cercaria and adult worms and is the recommended drug for schistosomiasis treatment (Mutapi et al., 2017). In conventional treatment, the role of gut microbiome has been ignored. Furthermore, the gut microbiome may disturb treatment by drugs. Additionally, when a drug is unreasonably utilized, drug resistance gradually appears (Vale et al., 2017). Thus, the abundance and composition of schistosomiasis- related bacteria should be evaluated during the treatment process. The results will support the hypothesis that *S. japonicum* treatment modulates the gut microbiota composition under pathophysiological conditions. In the current study, the gut microbiome was evaluated with 16S rRNA sequencing on a small scale. In addition to bacteria, other microorganisms, including viruses, protists, and fungi, are also considered members of the gut microbiome. Furthermore, the life cycle of *S. japonicum*, including cercaria, schistosomulum, adults and eggs, is extremely complex. Different stages of *S. japonicum* will influence the microbiome of the skin, lungs, intestine, and mesentery, which recently has been considered a novel organ (Coffey and O'Leary, 2016). Thus, the sampling time and location will be fully considered in future studies. Moreover, metagenome sequencing for large-scale verification related to matched groups needs to be further validated.

In conclusion, our findings offer an insight into mouse gut microbial modulation after infection with *S. japonicum* cercaria. We observed an alteration in the gut microbial communities in response to *S. japonicum* treatment, which is more prominent in C57BL/6 than in BALB/c mice. The microbial community of the intestine could be used as a potential biomarker for the diagnosis of a *S. japonicum* infection and as an evaluation of the pathogenesis and progression of schistosomiasis.

## Ethics Statement

All animal treatments, including use and care, were performed in accordance with the recommendations of the Regulations for the Administration of Affairs Concerning Experimental Animals in China (11/14/1988). The animal experiment was approved by the Animal Research Advisory Committee of the Hubei University of Medicine under permit number HBMU-S20160414 and performed in the Collegial Laboratory Animal Center.

## Author Contributions

YZ, SY, BL, ZC, JY, HT, and JL contributed to the study concept and design, analysis and interpretation of data, and drafted the manuscript. YZ, SY, and JL performed the experiments. WL and JW contributed to the analysis and interpretation of data as well as the statistical analysis.

### Conflict of Interest Statement

The authors declare that the research was conducted in the absence of any commercial or financial relationships that could be construed as a potential conflict of interest.

## Supplementary Material

Supplementary Figure 1 General information regarding uninfected and infected mice. The weights of the body, liver and spleen and spleen size in each group from C57BL/6 and BALB/c mice were obtained.

Supplementary Figure 2 Species diversity and richness evaluation of mouse gut samples during infection. **(A)** Species accumulation curves. The vertical axis shows the number of observed species that are expected to be found, after sequencing the number of samples shown on the horizontal axis. Curvature toward the horizontal indicates the increased sampling effort required to find species when only rare species remain to be discovered. **(B)** Rarefaction curves. The vertical axis shows the number of observed species that are expected to be found after sampling the number of tags or sequences shown on the horizontal axis. Curvature toward the horizontal indicates the increased sequencing effort required to find species when only rare species remain to be discovered.

Supplementary Figure 3 Shared operational taxonomic unit (OTU) analysis of the different communities. Venn diagrams showing the unique and shared OTUs in the different groups and communities. Group and sample names refer to groups and samples as described in Table 1.

Supplementary Figure 4 Hierarchical clustering of feces samples by the Unweighted Pair-group Method with Arithmetic Mean (UPGMA) according to their unweighted UniFrac matrix. Bar charts show the relative abundance of the main bacterial phyla found in each of the groups. Phyla representing < 1% of the sequences in a group have been grouped as “other.” Group names refer to groups as described in **Table 1**.

Supplementary Table 1 Within- vs. Among-Group Dissimilarities analysis by Analysis of Similarities (ANOSIM).

Supplementary Table 2 Within- vs. Among-Group Dissimilarities analysis via Multi Response Permutation Procedure (MRPP).
