# Corrigendum: Alterations of the Mice Gut Microbiome via *Schistosoma japonicum* Ova-Induced Granuloma

**DOI:** 10.3389/fmicb.2019.00747

**Published:** 2019-04-09

**Authors:** Yanqing Zhao, Shuguo Yang, Bei Li, Wei Li, Jue Wang, Zongyun Chen, Jing Yang, Huabing Tan, Jian Li

**Affiliations:** ^1^Department of Human Parasitology, School of Basic Medical Science, Hubei University of Medicine, Shiyan, China; ^2^Department of Infectious Diseases, Renmin Hospital, Hubei University of Medicine, Shiyan, China; ^3^Department of Prevention and Control of Schistosomiasis, Jiangsu Institute of Parasitic Diseases, Wuxi, China

**Keywords:** *Schistosoma japonicum*, egg granulomas, gut microbiome, 16S rRNA, operational taxonomic units

In the published article, there was an error in affiliation 1. Instead of “Department of Human Parasitology, School of Basic Medical Science, Shiyan, China,” it should be “Department of Human Parasitology, School of Basic Medical Science, Hubei University of Medicine, Shiyan, China.”

The authors apologize for this error and state that this does not change the scientific conclusions of the article in any way.

