# Supplementary figures and images for: Alterations of the Mice Gut Microbiome via Schistosoma japonicum Ova-Induced Granuloma
Source: Front Microbiol. 2019 Mar 5;10:352. doi: 10.3389/fmicb.2019.00352 (PMC6411663; doi:10.3389/fmicb.2019.00352)

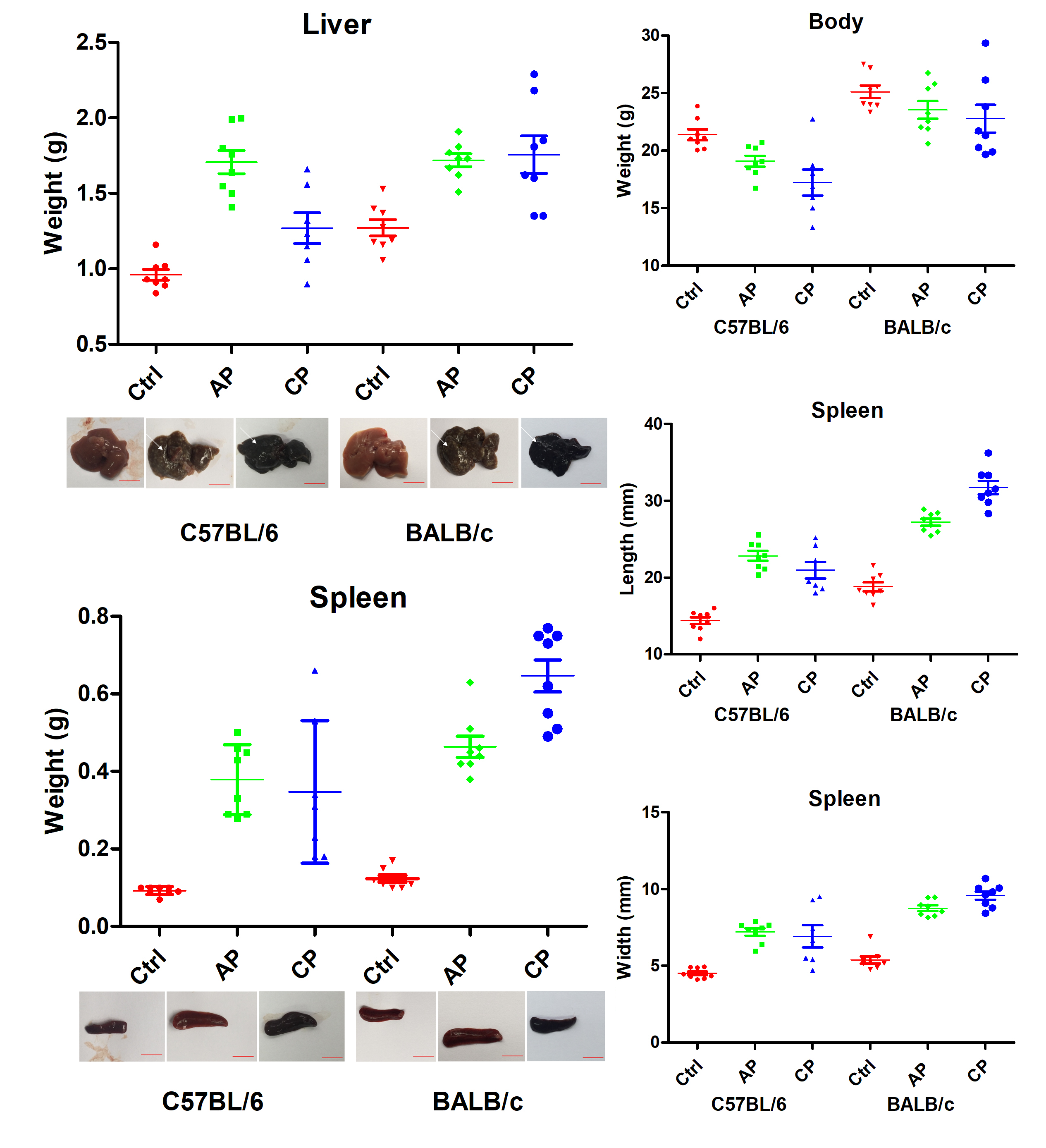

Supplement: Supplementary Figure 1 — General information regarding uninfected and infected mice. The weights of the body, liver and spleen and spleen size in each group from C57BL/6 and BALB/c mice were obtained. [file Image_1.TIF]

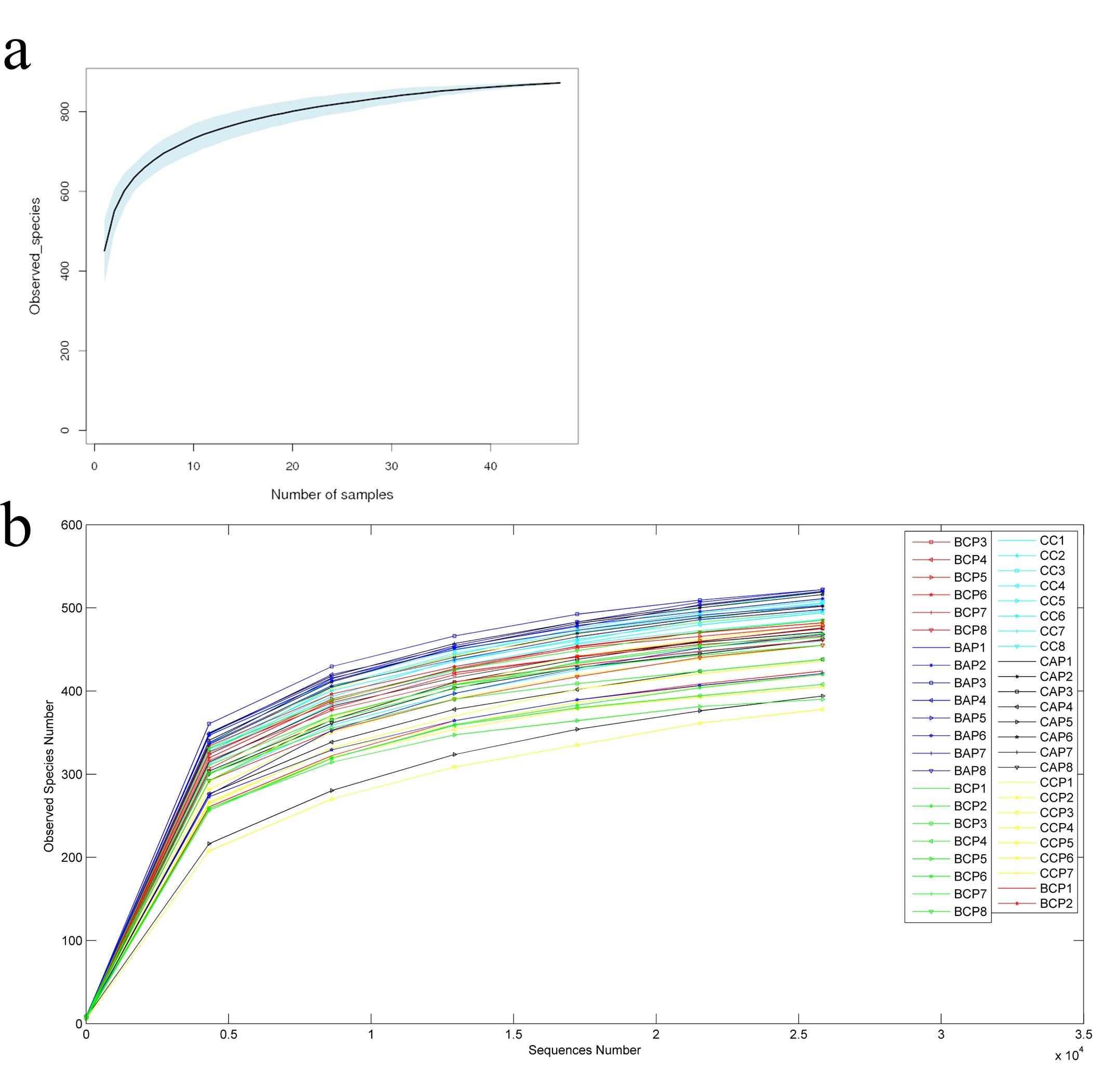

Supplement: Supplementary Figure 2 — Species diversity and richness evaluation of mouse gut samples during infection. (A) Species accumulation curves. The vertical axis shows the number of observed species that are expected to be found, after sequencing the number of samples shown on the horizontal axis. Curvature toward the horizontal indicates the increased sampling effort required to find species when only rare species remain to be discovered. (B) Rarefaction curves. The vertical axis shows the number of observed species that are expected to be found after sampling the number of tags or sequences shown on the horizontal axis. Curvature toward the horizontal indicates the increased sequencing effort required to find species when only rare species remain to be discovered. [file Image_2.JPEG]

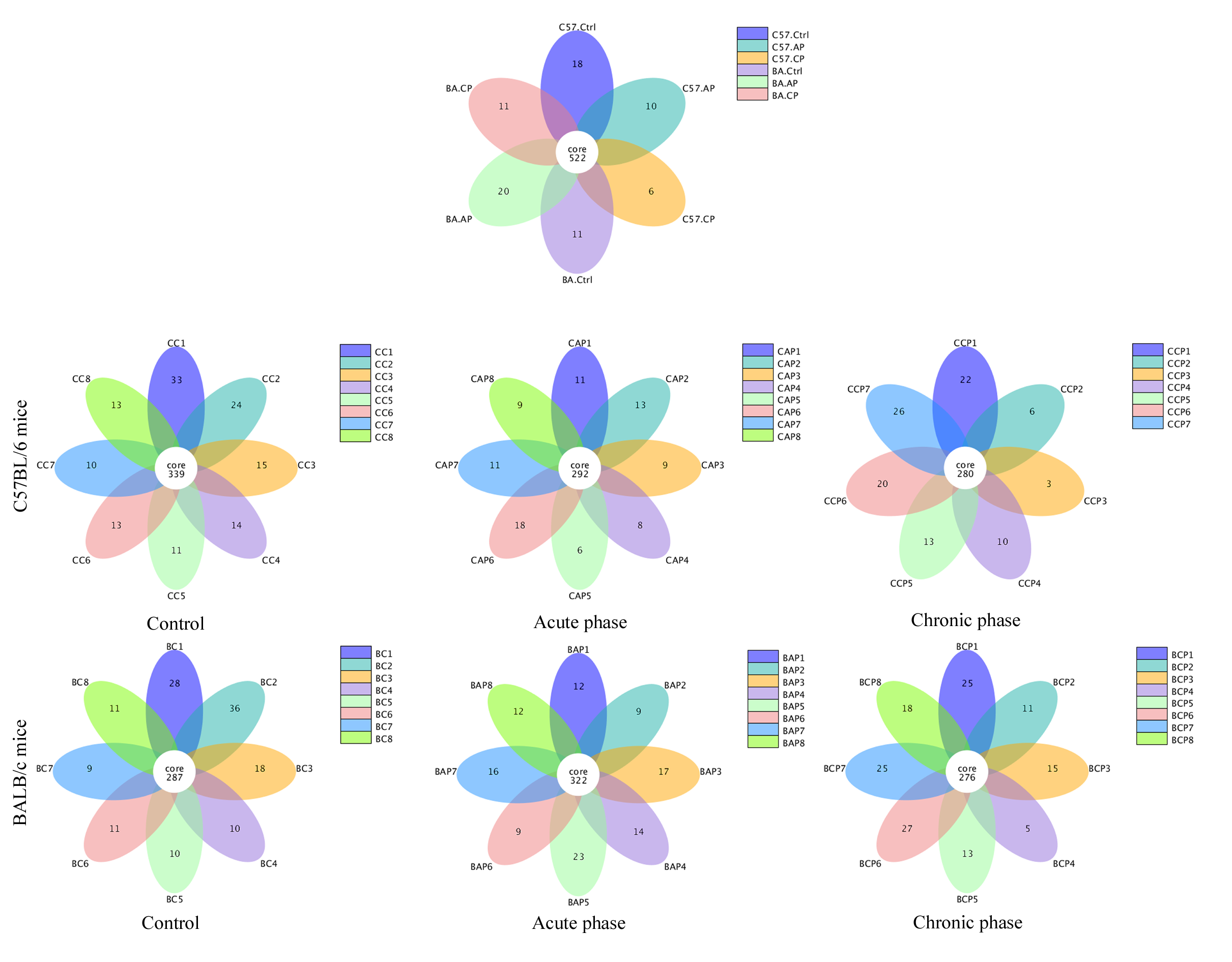

Supplement: Supplementary Figure 3 — Shared operational taxonomic unit (OTU) analysis of the different communities. Venn diagrams showing the unique and shared OTUs in the different groups and communities. Group and sample names refer to groups and samples as described in Table 1. [file Image_3.TIF]

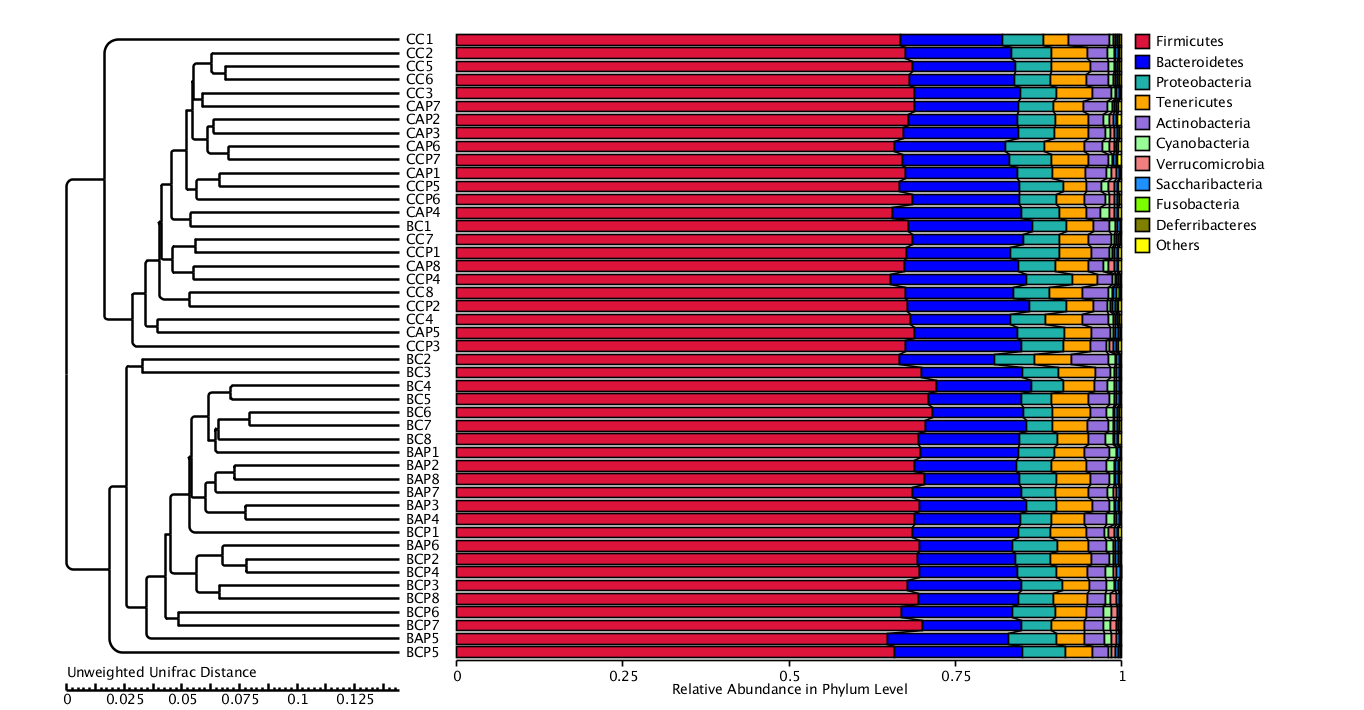

Supplement: Supplementary Figure 4 — Hierarchical clustering of feces samples by the Unweighted Pair-group Method with Arithmetic Mean (UPGMA) according to their unweighted UniFrac matrix. Bar charts show the relative abundance of the main bacterial phyla found in each of the groups. Phyla representing < 1% of the sequences in a group have been grouped as “other.” Group names refer to groups as described in Table 1. [file Image_4.PNG]
